# Population-Level Dynamics and Community-Mediated Resistance to Antimicrobial Peptides

**DOI:** 10.3390/biom15091319

**Published:** 2025-09-15

**Authors:** Theresia Mekdessi, Aracely Devora, Sattar Taheri-Araghi

**Affiliations:** Department of Physics and Astronomy, California State University at Northridge, Northridge, CA 91330, USA

**Keywords:** antimicrobial peptides, community-level resistance, sequestration, phenotypic heterogeneity, inoculum effect

## Abstract

Antimicrobial peptides (AMPs) are crucial components of innate immunity and promising leads for new anti-infective therapies, prized for their broad-spectrum activity and membrane-disruptive mechanisms. However, traditional models of antimicrobial action and resistance often focus on single-cell responses or genetically encoded resistance, overlooking the complex collective behaviors of bacteria at the population level. A growing body of evidence indicates that bacterial communities can profoundly influence AMP efficacy through emergent, community-level resistance mechanisms. In this review, we examine how population-level dynamics and interactions enable bacteria to withstand AMPs beyond what is predicted by cell-autonomous models. We first describe the mechanisms of peptide sequestration by bacterial debris, dead cells, outer membrane vesicles, and biofilm matrix polymers, which diminish the concentration of active peptide available to kill neighboring cells. We then analyze how population-level traits—including inoculum effects, phenotypic heterogeneity, and persister subpopulations—shape survival outcomes and promote regrowth after treatment. Cooperative processes such as protease secretion further enhance communal defenses by coordinating or amplifying protective responses. Beyond cataloging these mechanisms, we highlight recent advances in microfluidic tools, single-cell imaging, and biophysical modeling that reveal the spatial and temporal dynamics of AMP action in structured populations. Collectively, these insights show how bacterial communities absorb, neutralize, or delay AMP activity without genetic resistance, with important implications for therapeutic design and the evaluation of AMP efficacy.

## 1. Introduction

Antimicrobial peptides (AMPs) play a central role in host defense and are increasingly explored as next-generation antimicrobials against drug-resistant pathogens [1,2,3,4,5]. These molecules, often cationic and amphipathic, target bacteria by permeabilizing membranes or disrupting critical cellular processes, leading to rapid bacterial death [6,7,8]. At the molecular and single-cell level, a wealth of research has elucidated how AMPs interact with bacterial membranes and how individual bacteria can evade or resist killing (for example, through membrane charge modifications or efflux pumps) [9,10,11,12]. However, bacteria rarely exist in isolation; in natural and clinical settings they form dense populations and biofilms where cell–cell and cell–environment interactions can dramatically alter the outcome of antimicrobial challenges [13,14,15,16,17]. This difference suggests that traditional cell-level models of AMP action may not fully capture the complex behavior of AMPs in bacterial communities.

Recent research has increasingly revealed that bacterial populations mount defenses as communities, exhibiting collective strategies that go beyond the capabilities of individual cells. One well-documented phenomenon is the inoculum effect, where the apparent potency of antimicrobials, including AMPs, decreases as the initial population size increases [18,19,20,21]. This suggests that dense populations can buffer or neutralize AMPs through community-level interactions. The collective defense mechanisms against AMPs are discussed in terms of both passive and active strategies. Passively, dead cells and extracellular materials are found to act as sinks, reducing the bioavailability of AMPs [18,19,22,23]. Bacteria also produce protective components like outer membrane vesicles and biofilm matrices that physically limit AMP penetration [24,25,26,27]. Actively, cells communicate via signaling pathways to coordinate communal defenses, such as triggering efflux pumps or inducing biofilm formation in response to population cues or damage signals [28,29,30]. Furthermore, phenotypic heterogeneity within bacterial populations, which arises from intrinsic stochastic fluctuations in gene expression and metabolism as well as external environmental factors such as nutrient availability and stress signals, contributes to their resilience. Even in genetically identical cells, variation in growth states and membrane properties leads to a spectrum of susceptibilities to antibiotics [31,32,33]. Subpopulations such as persisters or heteroresistant cells can survive treatment and later regrow [12,34,35].

In light of these findings, it is evident that focusing solely on cell-intrinsic resistance mechanisms provides an incomplete picture of how bacteria withstand AMPs. Population-level dynamics—involving both physical factors (such as peptide sequestration and spatial structuring) and biological interactions (including cell–cell signaling and phenotypic heterogeneity)—play a pivotal role in determining treatment outcomes of AMP [15,18,28,33,36]. Understanding these collective mechanisms is essential for microbiologists and biophysicists seeking to accurately assess the real-world efficacy of antimicrobial peptides.

Here, we present a comprehensive review of how bacterial populations and communities resist AMPs through collective and emergent strategies. We begin by examining mechanisms of peptide sequestration, including absorption by dead cells, outer membrane vesicles, and biofilm matrix polymers. We then explore how population-level traits—such as the inoculum effect, phenotypic heterogeneity, and the presence of persister subpopulations—modulate AMP efficacy. The review also highlights recent advances in microfluidic techniques and mathematical modeling that illuminate the spatial and temporal dynamics of AMP action in dense or structured populations. Together, these insights underscore the importance of considering bacterial community behavior when evaluating AMP potency and guide future approaches to antimicrobial design and deployment.

## 2. Materials and Methods

Generative AI (ChatGPT, version 4o, July 2025) was used during the preparation of this review article to correct grammar and typographical errors, as well as to improve wording, clarity, and overall presentation of the English language.

## 3. Community-Mediated Resistance to Antimicrobial Peptides

### 3.1. AMP Sequestration and Reduced Bioavailability in Bacterial Populations

#### 3.1.1. Sequestration of AMPs by Dead Cells

A key mechanism by which bacterial communities tolerate AMPs is the sequestration of these molecules by dead or dying cells. Upon exposure to AMPs, not all bacteria are lysed simultaneously; instead, single-cell studies have shown that certain subpopulations—often those in vulnerable physiological states—die first and absorb a large amount of the peptides. This behavior has been particularly well characterized for the human cathelicidin LL-37: when *E. coli* is exposed to LL-37, the population frequently appears to split into two functional groups—cells that become permeabilized and absorb peptide, and others that survive and continue to grow in the same environment [18,19].

**Figure 1:**
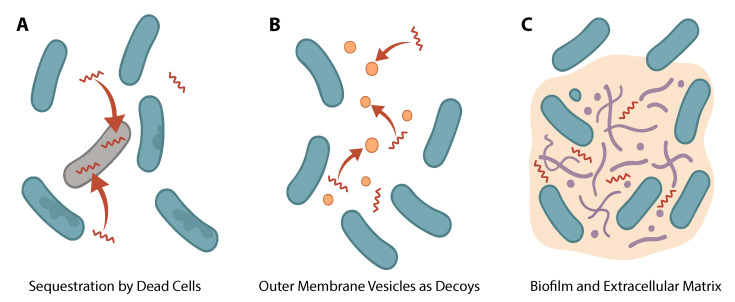
Mechanisms of antimicrobial peptide (AMP) sequestration by bacterial communities. (**A**) Dead or dying cells passively absorb and bind AMPs, acting as peptide sinks that reduce the concentration of active peptides available to kill neighboring cells. (**B**) Gram-negative bacteria release outer membrane vesicles (OMVs) that act as decoys, sequestering AMPs through electrostatic interactions with lipopolysaccharides and membrane proteins. (**C**) In biofilms, extracellular matrix components such as extracellular DNA and polysaccharides bind AMPs, slowing their diffusion and creating spatial gradients that shield cells embedded deep within the matrix. Together, these mechanisms contribute to community-level tolerance and reduced AMP efficacy.

Importantly, these subpopulations are not genetically or phenotypically distinct prior to exposure. As schematically shown in Figure 1A, the permeabilized cells act as sacrificial “sinks” as soon as AMPs disrupt their membranes, thus negatively charged intracellular molecules such as DNA and ribosomal proteins start interactions with AMPs. These anionic components bind cationic AMPs through non-covalent interactions, effectively trapping the peptides within the compromised cells and reducing the concentration of active AMPs available to kill other bacteria [18,19,37]. This mechanism is best described as a passive rather than an active strategy; it does not reflect an evolved resistance trait based on cell–cell communication, but rather a consequence of AMP electrostatic interactions with intracellular components.

This phenomenon is observed at sublethal peptide concentrations, where not all cells are killed. Those that absorb and neutralize the AMPs protect the remaining population, allowing survivors to regrow once the active peptide is depleted. This may create the appearance of persistence or a prolonged lag phase. However, the observed regrowth simply reflects the surviving fraction of cells resuming normal division, rather than a true quiescent state. At higher AMP concentrations, this effect is overwhelmed and near-complete killing is achieved. The sequestration process also helps explain the inoculum effect, where higher initial cell densities improve overall survival by providing more sacrificial targets to bind AMPs [18,20].

#### 3.1.2. Outer Membrane Vesicles as Decoys

Gram-negative bacteria, in particular, can release outer membrane vesicles (OMVs) that bind and neutralize AMPs before these peptides reach live cell targets [24]. As schematically shown in Figure 1B, OMVs are nanoscale blebs of outer membrane that carry lipopolysaccharide (LPS), phospholipids, and outer membrane proteins. Since AMPs often target LPS-rich membranes, OMVs function as decoys to attract and sequester AMPs in the surrounding environment [24,38]. Studies have found that sublethal exposure to certain AMPs triggers bacteria to increase OMV production [39,40]. For instance, *E. coli* and other Gram-negatives exposed to sublethal AMPs (such as CATH-2 or PMAP-36) dramatically upregulate OMV release, presumably as a stress response to envelope damage [39]. These OMVs can bind AMPs in solution: the presence of purified OMVs has been shown to raise the minimum bactericidal concentration of AMPs needed to kill bacteria, confirming that OMVs protect cells by acting as AMP sponges [24,39,40,41].

This mechanism is largely biophysical. The electrostatic attraction between the cationic peptides and the anionic LPS and proteins on OMVs binds the AMPs to the vesicles [24,41]. Once an AMP is bound to or incorporated into an OMV, it is effectively segregated away from bacterial cells; some OMVs may subsequently be shed or degraded, permanently removing the peptide [38,40]. In addition, OMVs can sometimes carry proteases or enzymes that further inactivate AMPs [38]. This decoy strategy has been noted to contribute to innate resistance: a hyper-vesiculating mutant of *E. coli* survived polymyxin B (a cationic peptide antibiotic) better than wild-type, and adding exogenous OMVs to bacterial cultures immediately reduced AMP killing [24,41]. Notably, this form of protection is cooperative at the population level—OMVs released by any cells in the community can shield others in the vicinity [38]. Even pathogenic species like *Acinetobacter baumannii* and *Pseudomonas aeruginosa* are shown to exploit OMV release as a resistance strategy to polymyxin [38,42]. The implication is that therapies based on AMPs might face resistance in clinical settings due to abundant OMV production, especially in infections with high bacterial loads [39,42].

Several genes in different organisms have now been identified that link to OMV production. In *Salmonella*, the PhoPQ-regulated pagC gene promotes vesiculation, with OMVs enhancing survival under immune attack [43,44]. In *Vibrio cholerae*, bap1 and ompT are found to be essential for loading protective cargo into OMVs [40]. In *enterohemorrhagic E. coli*, ompT likewise facilitates OMV-mediated binding of LL-37, increasing resistance [45]. Finally, mutations in envelope-linked genes such as tolR and degS drive hyper-vesiculation, with released OMVs acting as decoys that are found to neutralize polymyxin B [46]. Together, these findings demonstrate that both regulatory and structural genes are involved in controlling OMV synthesis during AMP stress.

#### 3.1.3. Biofilm Matrix and Extracellular Polymers

Bacteria in biofilms are embedded in a self-produced matrix composed of polysaccharides, proteins, lipids, and extracellular DNA [47]. This matrix may act as a physical barrier to diffusion and may also sequester antimicrobial agents that invade bacteria [26]. Polyanionic components of the matrix, such as DNA and acidic exopolysaccharides, can bind cationic AMPs via electrostatic interactions, preventing the peptides from freely reaching the bacteria (Figure 1C). The addition of exogenous DNA has been shown to protect *P. aeruginosa* biofilms from cationic antimicrobials by this mechanism [48,49]. Similarly, capsular polysaccharides and exopolysaccharides provide a charge-based repellent effect: a classic study demonstrated that a *Klebsiella* strain with its capsule intact had up to 1000-fold higher resistance to cationic AMPs compared to a non-capsulated mutant, due to the capsule’s ability to prevent peptide access to the membrane [9]. Matrix-associated proteins can also contribute to AMP sequestration. The group A *Streptococcus* M1 protein, for instance, is secreted and embeds in the bacterial surface and matrix, where it directly binds and neutralizes host defense peptides, essentially acting as a peptide “sponge” and preventing AMP-mediated membrane disruption [50].

Triggering biofilm matrix production is a common bacterial strategy upon sensing stress: sub-inhibitory AMP exposure has been shown to induce the production of curli fibers and colanic acid polysaccharide in *E. coli*, which are matrix components that then shield the cells from subsequent peptide attack [51]. Nutrient limitation can induce enhanced extracellular polymeric substance (EPS) secretion, which supports aggregation and provides alternative nutrient sources under starvation [52,53]. Nutrient shifts drive enhanced EPS production, which reinforces the biofilm’s structural framework [54,55]. Such nutrient-regulated matrix accumulation generates stratified microenvironments that maintain metabolically diverse subpopulations [56]. Studies in *extremophiles* further show that cells prioritize EPS synthesis over other polymers, emphasizing matrix production as a key survival strategy [52]. Nutrient-driven EPS production plays a central role in stress resilience and provides a basis for AMP resistance [52,53,54,55,56].

### 3.2. Phenotypic Heterogeneity and Community-Level Resistance

#### 3.2.1. Persisters and Phenotypic Variants

Beyond single-cell resistance, bacterial populations exhibit phenotypic heterogeneity that leads to varying susceptibilities within the community [31,57]. Differences in growth states, gene expression, and stress responses give rise to several forms of community-mediated resistance, including “persistence.” Persister cells are a small subset of the population, typically found in slow-growing or nongrowing states, that are temporarily tolerant to killing by antibiotics [31,34]. These cells are not genetically resistant; once the antibiotic is removed, they can regrow a population with the original sensitivity. However, they survive transient exposure to otherwise lethal treatments by entering a tolerant, dormant state [31,57]. Persister formation is considered a form of phenotypic bet-hedging that ensures some cells survive sudden stress [33].

**Figure 2:**
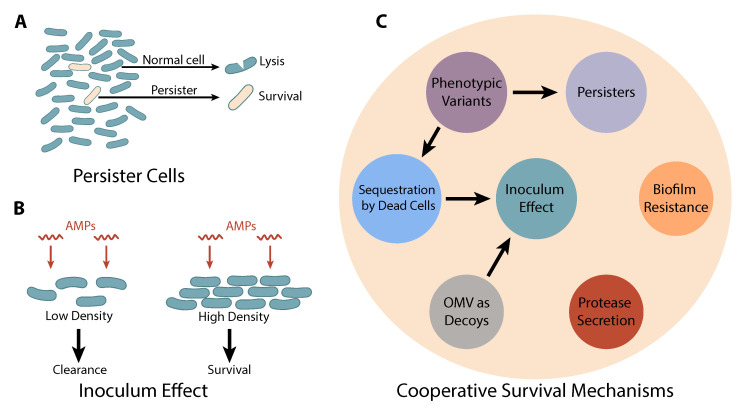
Phenotypic heterogeneity and community-level resistance to antimicrobial peptides (AMPs). (**A**) Illustration of persister cells: a subpopulation of dormant or slow-growing cells that tolerate AMP exposure and can repopulate after the treatment subsides. (**B**) Graphical representation of the inoculum effect, showing that higher bacterial densities exhibit increased survival at the same AMP concentrations due to collective buffering mechanisms. (**C**) Schematic of cooperative survival strategies within bacterial communities. The diagram highlights the interrelated roles of dead cell AMP sequestration, outer membrane vesicle (OMV) decoy production, biofilm matrix binding, persister cell formation, and protease secretion. Arrows and overlaps illustrate how these mechanisms collectively contribute to community-level resistance, with multiple layers of protection emerging from both passive and active processes.

There is evidence that persister-like cells can also be induced experimentally to tolerate AMPs as schematically shown in Figure 2A. Recent work has shown that *E. coli* populations primed with sublethal doses of AMPs exhibited an increased fraction of survivors upon subsequent high-dose challenge, implying induction of a persister-like state [51]. In that study, initial AMP exposure triggered the upregulation of protective envelope components (such as curli and colanic acid) and other stress pathways, leading to more cells capable of “waiting out” the attack. These persister-like cells can then regrow once the AMP is depleted or degraded. Heteroresistance is a related, broader phenomenon: even without prior priming, a clonal population can display a range of susceptibilities, with a small fraction of cells surviving antibiotic treatment [58]. Such heteroresistant subpopulations may arise from stochastic expression differences—for example, random high expression of efflux pumps or stress-response regulators in a few cells [33]. In the context of AMPs, heteroresistance has been documented where a minority of cells survive AMP exposure due to transiently elevated protective factors; when those survivors multiply, the resulting population again contains only a small fraction of highly tolerant cells [59]. This non-uniform behavior can drastically affect treatment outcomes, as the surviving fraction can seed regrowth and prolong infection.

However, the nature of persistence under AMP treatment remains an open question. Because AMPs primarily act through membrane disruption—rather than targeting growth-related processes—it is unclear whether dormancy provides the same protection observed with conventional antibiotics. Recent high-resolution single-cell studies have shown that antimicrobial peptides such as LL-37 remain potently active against nongrowing *E. coli*, albeit with slower kinetics [60]. This suggests that dormancy alone does not ensure survival under AMP challenge, and that persistence mechanisms in AMP-exposed populations may differ fundamentally from those associated with classical antibiotics.

#### 3.2.2. The Inoculum Effect

The ability of bacteria to withstand AMPs often increases with population size—a phenomenon known as the inoculum effect (Figure 2B) [18,19,20,61]. In practical terms, a higher initial bacterial density can require a higher concentration of AMP to achieve the same killing effect. Several of the mechanisms discussed in this review contribute to the inoculum effect. Sequestration by dead cells and OMVs are two clear contributors: the more bacteria present, the more potential decoys and absorptive surfaces exist to soak up the peptide, effectively diluting the attack [18,19,24]. Enzyme-mediated AMP degradation (e.g., by secreted proteases or peptidases) is also more effective in larger populations where enzyme levels accumulate [26]. Another contributor is the dynamic survival response of the population—bacteria do not die instantaneously upon AMP exposure, and during the time when some are succumbing, others may mount stress responses or enter tolerant states [51]. At high cell densities, this leads to an extended battle in which an initial fraction of cells is killed, removing a portion of the AMP via sequestration, while the remaining cells, if not fully eliminated, may recover and resume growth once the local AMP concentration falls.

Experiments with Gram-negative bacteria and cationic peptides have shown that with a low inoculum, an AMP can sterilize the culture, but with a high inoculum, the same dose only causes a temporary drop in viable cell count followed by regrowth of survivors [62]. Different experimental approaches have been used to test the inoculum effect. Snoussi et al. employed a modified microplate assay across inocula ranging from $0.5\times 10^{6}$ to $6\times 10^{6}$ CFU/mL, revealing that even under dilute conditions (cells ∼50 μm apart), MIC values rose with inoculum size [18]. Loffredo et al., using a classical broth microdilution assay across a much broader range of cell densities (50 to $10^{8}$ CFU/mL), demonstrated that MICs can increase up to 100-fold at high densities [20].

Mathematical models of AMP action have successfully reproduced inoculum effects by incorporating density-dependent sequestration terms or subpopulations of “binding” cells [18,63,64]. Importantly, the inoculum effect is not merely an in vitro artifact. In infections, large bacterial loads—such as those found in abscesses or biofilm-covered surfaces—often display reduced susceptibility to host AMPs or AMP-based therapeutics, requiring higher doses or combination strategies for effective clearance.

### 3.3. Other Cooperative Survival Mechanisms

Many of the processes discussed above are inherently cooperative, meaning they emerge from the interactions or contributions of multiple cells. Some of them overlap with each other in the context of how they contribute to observable, emergent effects at the population level. The relative contributions of these mechanisms to emergent resistance phenomena are summarized in Table 1 and schematically shown in Figure 2C.

Some of these processes are passive, like the passive protection provided by dead cells or OMVs, which do not require coordinated action. Note that there are also many observations of active cooperation of bacterial communities against antibiotics, such as quorum-sensing regulated responses or the necrosignaling [28]. An example of active cooperation against AMPs is the secretion of public goods such as extracellular proteases that inactivate host defense molecules.

Several pathogens, including *P. aeruginosa*, *S. enterica*, and *Staphylococcus aureus*, produce proteases like elastase (AprA), PgtE, and aureolysin, respectively, that cleave the AMP LL-37, resulting in a loss of its antimicrobial activity and enabling increased bacterial survival in hostile environments [65,66,67]. These proteases act on specific peptide bonds within LL-37’s active region, and their presence in complex fluids like wound exudates or plasma has been shown to render LL-37 ineffective [65,67]. In *S. aureus*, aureolysin-producing strains are significantly more resistant to LL-37 than their non-producing counterparts, demonstrating the functional relevance of protease secretion [68]. Similarly, in *Salmonella typhimurium*, the omptin family protease PgtE promotes resistance to alpha-helical AMPs such as C18G [66]. Since these proteases are secreted into the extracellular milieu, they operate as public goods—their production is metabolically costly for the individual cell, but they provide communal protection by detoxifying AMPs before they reach the bacterial surface.

From an evolutionary perspective, these collective tolerance mechanisms are worrying because they extend the survival window of bacteria under antimicrobial treatment, which, in turn, provides more opportunity for true genetic resistance mutations. Tolerance and persistence are known to be stepping stones to resistance: by surviving longer, bacteria increase the chance to acquire mutations or mobile elements that confer permanent resistance [69]. Community-mediated resistance, thus, has clinical ramifications (discussed next), as it can be a precursor to treatment failure and the emergence of hardier strains.

## 4. Clinical and Biophysical Implications

### 4.1. Implications for Therapy and Rational Design of Peptide-Based Antimicrobials

Understanding population-level dynamics of AMP resistance is not only of importance in basic research but has direct clinical relevance. In real infections, bacteria rarely exist as isolated planktonic cells; instead, they form dense communities (biofilms, microcolonies, swarms) with spatial structure, cell–cell interactions, and the ability to mount collective defenses as described.

Community-level resistance mechanisms mean that an infection with a high bacterial load or biofilm organization may withstand host innate immune attacks or AMP-based drugs even if the bacteria are “susceptible” in standard planktonic lab tests. The innate immune system relies on producing AMPs at infection sites [70], but if the bacterial load is high, those peptides might be rapidly sequestered by bacterial debris, matrix, or dead cells. The host would need to continuously produce fresh AMPs to overcome the loss to these sinks. This insight emphasizes the importance of AMP concentration dynamics in vivo: transient bursts of AMP might knock down the bacterial population but not clear it, after which the surviving community can regrow [51,71]. Clinically, this might manifest as a recurrence or persistence of infection after an initial response. It also suggests that simply increasing the dose of an AMP-based therapeutic could face diminishing returns if the extra peptides are just absorbed by additional dead cells or biofilm material [72,73]. Instead, combination strategies might be required—for example, co-administering matrix-degrading enzymes or inhibitors of OMV biogenesis to prevent the bacteria from using these decoys [74].

Another implication is in diagnostics and susceptibility testing: traditional MIC tests that inoculate a relatively low number of bacteria in broth or on Petri dishes may not reveal an inoculum effect, whereas in the patient, the bacterial population could be large and far more tolerant. Thus, there is growing recognition that drug screening and testing should account for community context (e.g., testing activity against bacteria in biofilm or at high densities) to better predict clinical outcomes [73,75]. From a public health perspective, community-mediated tolerance is a concern because it can promote the development of true resistance [76]. Surviving populations after AMP exposure could acquire mutations in, say, lipid A modification enzymes or efflux pumps, leading to heritable resistance. This has been shown in laboratory experiments where repeated cycles of incomplete killing (tolerant subpopulations surviving) led to selection of strains with permanent resistance mechanisms [51]. Therefore, therapies that can achieve rapid and complete eradication of bacteria are desired to avoid creating a breeding ground for resistance evolution [76].

### 4.2. Broader Implications: Overcoming Community-Level Resistance

While antimicrobial resistance is often tackled at the single-cell level, community-level resistance represents a more diffuse and complex barrier. It arises from collective behaviors that fundamentally differ from single-cell resistance or tolerance mechanisms such as point mutations or enzymatic adaptations. These are emergent properties of the group, making them harder to circumvent with a single chemical modification or dose increase.

Overcoming community-level defenses requires system-level strategies, often indirect, that reshape the environment or target multiple levers at once. Simply increasing peptide concentration can yield diminishing returns if the extra AMP is absorbed by cellular debris or trapped in a matrix. Instead, researchers are exploring multi-pronged approaches, such as disrupting decoy mechanisms, dispersing biofilms, blocking vesicle formation, or interfering with bacterial communication [74].

Promising advances have come from synergistic combinations. Pairing AMPs with conventional antibiotics can increase membrane permeability, enabling deeper antibiotic penetration and improved killing even in biofilms [77]. Host molecules such as histones provide another striking example: histone H2A can enter through AMP-created pores and disrupt both membrane potential and chromosome organization, producing a lethal synergy that individual agents cannot achieve [77,78]. These examples underscore that collective problems require collective solutions, where multiple modes of action converge on the bacterial community.

Ultimately, strategies to overcome community-mediated tolerance are still at an early stage. They are more like guiding stars than established roads, highlighting that the path forward will require broader thinking that integrates molecular design, delivery systems, diagnostics, and even ecological considerations. Translating these insights into clinical practice will depend on treating infections not just as isolated cells but as organized populations, where disarming communal defenses may be as crucial as killing individual bacteria.

## 5. Models and Tools to Probe Community Resistance

### 5.1. Biophysical Modeling and Theoretical Insights

To quantitatively capture the complex dynamics of AMP action, researchers have developed mathematical models that integrate microbiological observations with concepts from statistical physics. These population dynamics models include terms for bacterial growth, death, persister formation, peptide binding, and AMP inactivation. For example, Snoussi et al. introduced a two-state model distinguishing AMP-absorbing dead cells from growing live cells. This framework explained population bifurcation under LL-37 treatment and quantitatively accounted for the inoculum effect on the apparent MIC [18].

Recent theoretical work has further refined our understanding of AMP selectivity through mechanistic models. Schefter et al. demonstrated that peptide trapping significantly amplifies the cell-density dependence of both the MIC and the minimum hemolytic concentration (MHC), thereby enhancing the inoculum effect [64]. Their model showed that the number of peptides absorbed per cell often exceeds $10^{7}$, with this absorption dominating over membrane-bound peptides in driving resistance at high cell densities [64]. Extending this framework, Lee et al. investigated competitive dynamics between bacterial and host cells, revealing that peptide trapping in host cells can artificially inflate noncompetitive selectivity (MHC/MIC) by up to two or three orders of magnitude when host cell density is high [63].

Together, these studies emphasize that peptide selectivity is not solely determined by single-cell or membrane-binding affinities, but also by contextual factors such as cell density and peptide sequestration.

### 5.2. Microfluidics and Single-Cell Analysis

Microfluidic devices, also referred to as lab-on-a-chip systems, have emerged as powerful tools to investigate bacteria–antibiotic interactions under realistic, precisely controlled conditions [79]. By miniaturizing culture environments and integrating controlled fluid flow, sensors, and microscopy, these platforms enable rapid antimicrobial susceptibility testing (AST) and effectively mimic key aspects of in vivo conditions that are often overlooked in standard bulk assays. For instance, Oates and Anastasiou developed a 3D-printed microfluidic device to evaluate doxycycline-releasing scaffolds under simulated oral infection conditions, employing continuous flow and dynamic pH fluctuations to replicate the oral cavity environment [80]. Such dynamic microfluidic experiments revealed drug release kinetics markedly different from static tests, demonstrating the potential of microfluidics to realistically model the complexity of infection environments.

Direct visualization of phenotypic heterogeneity in bacterial responses, particularly to AMPs, has been advanced using single-cell microfluidic setups. Small fluidic chambers [19] and patterned agarose gel microstructures [18], combined with time-lapse microscopy, have enabled tracking of individual bacterial cells under antibiotic stress. These systems have uncovered important phenomena, including the role of slow-growing or dormant subpopulations in community-level antibiotic tolerance [18,60]. Such single-cell platforms illustrate how population-level antibiotic responses emerge from the integration of heterogeneous single-cell behaviors, highlighting mechanisms of community survival and resistance that cannot be easily observed using bulk culture approaches.

Droplet-based microfluidic systems represent another significant advancement for high-throughput, rapid AST. These platforms compartmentalize individual bacteria into picoliter droplets, enabling massively parallel testing with drastically reduced detection times compared to conventional assays [81]. For example, Hsieh et al. combined droplet microfluidics with genetic probes to achieve pathogen identification and AST directly from clinical samples within approximately 30 min [81]. On larger scales, microfluidic biofilm models provide detailed spatial analysis of antibiotic-stressed bacterial communities. Bittihn et al. demonstrated that structured *E. coli* microcolonies in microfluidic traps develop nutrient gradients that mediate spatially heterogeneous antibiotic responses, thereby mirroring the protective stratification observed in clinical biofilms [82]. Collectively, these advanced microfluidic methodologies illuminate the complex, multiscale dynamics of bacterial antibiotic responses and help bridge simplified laboratory assays with the multifaceted reality of bacterial infections [83].

In parallel with microfluidic innovations, the hollow-fiber infection model (HFIM) has emerged as a powerful system for dynamic dose-response studies [84]. This platform uses semi-permeable fibers to create distinct compartments where bacteria are exposed to changing antimicrobial concentrations that closely mimic human pharmacokinetic profiles [85]. Unlike static assays, the HFIM enables long-term monitoring of bacterial populations under clinically relevant dosing regimens, providing insight into resistance emergence, persistence, and the optimization of combination therapies [86,87]. Together with microfluidics, the HFIM represents a complementary approach that bridges in vitro experimentation with clinically realistic treatment scenarios.

### 5.3. Single-Cell Raman Spectroscopy

Complementary to microfluidics, single-cell Raman spectroscopy (SCRS) provides a label-free, non-destructive optical method to probe the biochemical composition and metabolic state of individual bacteria [88]. By detecting vibrational fingerprints of cellular molecules, SCRS can uncover phenotypic heterogeneity within microbial populations, including tolerant or persister subpopulations that survive antimicrobial stress [89]. Notably, antibiotic-sensitive and resistant strains often display divergent spectral features, such as altered nucleic acid-to-protein ratios or differences in cell envelope composition, reflecting underlying physiological adaptations that confer resistance [90]. By extension, bacteria that have evolved or acquired resistance to antimicrobial peptides (AMPs) may likewise exhibit distinct Raman signatures, for example, through modifications in membrane lipids or wall polymers that reduce peptide activity. Such single-cell analyses provide a powerful means to identify resistant subpopulations and track their biochemical responses in situ, without the need for labels or extended culture steps.

A particularly powerful adaptation is the coupling of SCRS with stable isotope probing to assess single-cell metabolic activity. In Raman deuterium isotope probing (Raman-DIP), bacteria are exposed to heavy water (D_2_O), and metabolically active cells incorporate deuterium into newly synthesized biomolecules (e.g., lipids and carbohydrates), generating carbon–deuterium bonds detectable in the Raman spectrum [91]. This allows rapid phenotypic antimicrobial susceptibility testing: resistant or surviving cells maintain strong C–D peaks, whereas susceptible cells show little to no incorporation. Importantly, this metabolic readout occurs within minutes to hours, dramatically reducing the turnaround time compared to conventional culture-based assays. Moreover, Raman-based studies have directly visualized AMP-induced perturbations of bacterial membranes, with peptides such as magainin and cathelicidin-BF30 producing characteristic shifts in lipid vibrational bands that reveal distinct modes of membrane disruption [92]. These molecular-level insights not only confirm mechanisms of AMP activity but also enable differentiation of resistance strategies based on how bacterial cells remodel their envelopes. Taken together, SCRS and its isotope-based extensions provide a versatile toolkit for dissecting antibiotic and AMP resistance at single-cell resolution, enriching our understanding of heterogeneity, persistence, and the biochemical interplay between pathogens and antimicrobials.

## 6. Conclusions

This review highlights how antimicrobial peptide (AMP) action is profoundly shaped by the social and physical context of bacterial populations. We examined mechanisms including sequestration of peptides by dead cells, outer membrane vesicles, and biofilm matrix components; phenotypic heterogeneity and persister formation; inoculum effects; and cooperative behaviors such as protease secretion. Together, these processes allow bacterial communities to absorb, deflect, or outlast AMP exposure, often without genetic resistance, and explain why results from low-density planktonic assays can underestimate the challenge of clearing dense or structured infections.

These insights underscore that AMP development and testing must move beyond single-cell or broth-culture models. Integrating biophysical modeling, microfluidics, single-cell imaging, and spectroscopy can reveal when and how communal defenses arise. Future therapies will likely need to pair AMPs with strategies that disrupt these shared protections—such as vesicle or matrix inhibitors—to achieve rapid and complete clearance. By targeting both individual cells and the group-level behaviors that shield them, next-generation antimicrobials may overcome community-level tolerance and resistance and better meet the demands of clinical and environmental challenges.

## Figures and Tables

**Table 1:** Community-level mechanisms contributing to AMP resistance and their relative importance across emergent phenomena.

Mechanism/Factor	Community Survival	Delayed Killing	Inoculum Effect	Recurrence/ Regrowth
*Sequestration-based*				
AMP Sequestration (Dead Cells)	✓	✓	✓✓	—
Outer Membrane Vesicles (OMVs)	✓	✓	✓	—
Biofilm Matrix Sequestration	✓	✓✓	✓	✓
*Phenotypic/tolerance*				
Persisters/Phenotypic Variants	✓	—	—	✓✓
*Enzymatic inactivation (public goods)*				
Protease Secretion	✓	✓	✓	—

✓✓ denotes a particularly strong contribution; ✓ a meaningful contribution; — a minimal or no direct contribution.
